# Stigmasterol Exerts an Anti-Melanoma Property through Down-Regulation of Reactive Oxygen Species and Programmed Cell Death Ligand 1 in Melanoma Cells

**DOI:** 10.3390/antiox13030380

**Published:** 2024-03-21

**Authors:** Na-Ra Han, Hi-Joon Park, Seong-Gyu Ko, Phil-Dong Moon

**Affiliations:** 1 College of Korean Medicine, Kyung Hee University, Seoul 02447, Republic of Korea; nrhan@khu.ac.kr; 2 Korean Medicine-Based Drug Repositioning Cancer Research Center, College of Korean Medicine, Kyung Hee University, Seoul 02447, Republic of Korea; epiko@khu.ac.kr; 3 Department of Anatomy & Information Sciences, College of Korean Medicine, Kyung Hee University, Seoul 02447, Republic of Korea; acufind@khu.ac.kr; 4 Department of Preventive Medicine, College of Korean Medicine, Kyung Hee University, Seoul 02447, Republic of Korea; 5 Center for Converging Humanities, Kyung Hee University, Seoul 02447, Republic of Korea

**Keywords:** stigmasterol, melanoma, ROS, melanin, nitric oxide, PD-L1, CD8(+) T cell

## Abstract

Cancer immunotherapy as a promising anti-cancer strategy has been widely studied in recent years. Stigmasterol (STIG), a phytosterol, is known to have various pharmacological effects, including anti-inflammatory effects. However, the pharmacological role of STIG on melanoma immunotherapy has not been investigated. The present study demonstrates the anti-melanoma potency of STIG through the regulation of PD-L1 levels. The results reveal that STIG reduces reactive oxygen species (ROS) levels induced by hydrogen peroxide and increases glutathione levels decreased by α-MSH in B16F10 cells. Moreover, STIG significantly decreases melanin content and tyrosinase activities elevated by α-MSH. It also suppresses nitric oxide production induced by α-MSH. Additionally, STIG induces apoptosis with the up-regulation of PARP activation. STIG inhibits IFN-γ-induced PD-L1 expression and STAT1 phosphorylation levels. STIG also reverses the up-regulation of PD-L1 and phosphorylated STAT1 levels augmented by cisplatin, and STIG enhances CD8(+) T-cell-mediated cell death against B16F10 cells. These findings represent the first evidence of pro-apoptotic activity of STIG on melanoma cells through the down-regulation of ROS and PD-L1 pathways. Therefore, STIG may be an effective candidate for melanoma immunotherapy.

## 1. Introduction

Oxidative stress is implicated in most chronic diseases, including cancer [1]. Cancer initiation and progression are associated with oxidative stress, as it induces DNA damage and promotes cell proliferation [2]. Melanoma is considered to be the most aggressive skin cancer and is on the rise globally [3]. Oxidative stress is significantly involved in melanogenesis and melanoma formation [4]. Oxidative stress disrupts melanocytes’ homeostasis and triggers their malignant transformation [5]. Melanogenesis involves oxidation reactions and the production of hydrogen peroxide (H_2_O_2_), which further exacerbates oxidative stress on melanocytes [5]. Reactive oxygen species (ROS), a subset of free radicals, contribute to tumorigenesis [6]. Melanin generates higher levels of ROS, increasing melanoma susceptibility [4]. ROS also promote melanoma formation, and melanoma itself produces ROS, which in turn enhances resistance to chemotherapy [7,8]. Nitric oxide (NO), another type of free radical, is implicated in carcinogenesis [1]. In addition, as a melanogenesis-stimulating factor [9], NO plays a role in melanoma development [10].

Cancer immunotherapy as a promising anti-cancer strategy has been extensively studied [11]. The immune checkpoint proteins, programmed cell death protein 1 (PD-1), and programmed cell death ligand 1 (PD-L1) regulate the induction and maintenance of immune tolerance in the tumor microenvironment [12]. Oxidative stress and ROS have been shown to influence PD-L1 expression in cancer [13,14,15]. The PD-L1 expression inhibits T lymphocyte activation, and its binding to PD-1 on CD8(+) T cells induces immune evasion [13]. Thus, several agents targeting PD-L1 have been studied in an attempt to disrupt these checkpoints and activate T cell-based immunotherapy [16].

Phytosterols are bioactive substances occurring naturally in various plants. Stigmasterol (STIG), a phytosterol, is abundant in several plants [17], and it exhibits a range of pharmacological effects, including antioxidant [18], anti-cancer [19], and anti-inflammatory properties [20]. Regarding melanoma, it has been reported that STIG has cytotoxic effects on human malignant melanoma cell lines [21] and mouse malignant melanoma (B16F10) cell lines [22]. However, there are no studies on the modulatory effect of STIG on melanogenesis and immune checkpoints in melanoma.

The aim of the present study is to elucidate the role of STIG as a cancer immunotherapy agent. We investigated its antioxidant and anti-cancer properties in melanoma using B16F10 cells and the CD8(+) T cell line, CTLL-2 cells.

## 2. Materials and Methods

### 2.1. Preparation of STIG

STIG (Sigma-Aldrich Co., Ltd. St. Louis, MO, USA) was prepared by dissolving it in the minimum amount of ethanol and cell culture media, following established protocols [23,24], and the doses were determined based on previous research [18,20].

### 2.2. Cell Incubation and Treatment

B16F10 and A375 melanoma cell lines (Korean Cell Line Bank, Seoul, Republic of Korea) were incubated in DMEM medium containing 10% fetal bovine serum. Melan-a cells (a normal, non-tumorigenic mouse melanocyte cell line), which were kindly provided by Prof. Tae-Hoo Yi, were incubated in RPMI 1640 medium containing 10% fetal bovine serum and 12-O-Tetradecanoylphorbol 13-acetate (200 nM, Sigma-Aldrich Co., Ltd.). CTLL-2 cells (ATCC, Rockville, MD, USA) were incubated in RPMI 1640 medium containing 10% T-STIM with Con A (BD Biosciences, San Jose, CA, USA) and 10% fetal bovine serum in a 37 °C plus 5% CO_2_ environment. The cells were exposed to STIG (0.5–50 µg/mL), H_2_O_2_ (1 mM), α-melanocyte-stimulating hormone (α-MSH, 100 nM), cisplatin (20 μM), nutlin-3a (10 μM), rapamycin (20 μM), or IFN-γ (10 ng/mL).

### 2.3. Analysis of Intracellular ROS

B16F10 cells were exposed to STIG for 1 h and then incubated with H_2_O_2_ for an additional 30 min at 37 °C. The cells were incubated with 20 μM H2DCFDA (Abcam, Cambridge, MA, USA) at 37 °C for 30 min. The absorbance was determined by using a fluorescence microplate reader at 485/535 nm. For confocal image analysis, the cells were treated with fluorescent mounting medium with DAPI and analyzed using Zeiss LSM800 fluorescent confocal microscopy (Carl Zeiss, Oberkochen, Germany).

### 2.4. Measurement of Glutathione (GSH) Content

B16F10 cells were exposed to STIG for 1 h and then incubated with α-MSH for 72 h. The total amount of intracellular GSH was analyzed with a GSH assay kit (DoGenBio Co., Ltd., Seoul, Republic of Korea) based on previous reports [25,26]. The results were expressed as percentages of increase over α-MSH-treated cells (control).

### 2.5. Melanin Content and Tyrosinase Inhibition Assay

The melanin content and tyrosinase inhibition assay were conducted according to modified methods [27,28]. The B16F10 cells and melan-a cells were exposed to STIG or α-MSH in 6-well plates. To measure the melanin content for STIG, the quantified pellets were lysed with 1 N NaOH containing 10% dimethyl sulfoxide for 1 h at 80 °C. The melanin content was then analyzed using a microplate reader at 405 nm. To measure tyrosinase inhibition for STIG, the cells were lysed in 1% Triton X-100 for 2 h, and the quantified pellets were treated with 10 mM L-DOPA for 30 min. The tyrosinase activity was analyzed using a microplate reader at 475 nm.

### 2.6. MTT Cell Viability

MTT solution (5 mg/mL) was added to B16F10 cells, melan-a cells, and A375 cells at 37 °C. The formazan dissolved in dimethyl sulfoxide was analyzed with a microplate reader at 540 nm.

### 2.7. Immunoblot Analysis

The protein quantification from B16F10 cells extracted by a RIPA buffer was determined using a BCA assay kit. Equal protein samples were electrophoresed using SDS-polyacrylamide gel and transferred to nitrocellulose membranes. The blocked membranes were incubated with primary antibodies (tyrosinase, tyrosinase-related protein-1 (TYRP-1), inducible NOS (iNOS), PARP, PD-L1, phosphorylated signal transducer and activator of transcription 1 (phospho-STAT1, Tyr701), STAT1, GAPDH, and tubulin, Santa Cruz Biotechnology, Inc., Dallas, TX, USA) and horseradish peroxidase (HRP)-conjugated secondary antibodies. Immunoreactive protein expression was visualized using ECL chemiluminescence reagents (DoGenBio Co., Ltd.).

### 2.8. NO Measurement

NO levels were assessed by analyzing nitrite concentration using Griess reagent as described in the literature [29]. Supernatants from STIG-treated B16F10 cells were added to 50 μL of Griess solution A (0.1% N-(1-Naphthyl) ethylenediamine dihydrochloride) and 50 μL of solution B (1% sulfanilamide prepared in 5% phosphoric acid). The optical density was then measured at 540 nm.

### 2.9. Immunofluorescent Staining

B16F10 cells were fixed with 4% paraformaldehyde, followed by permeabilization with 0.25% Triton X-100 for 10 min and blocking with 5% bovine serum albumin. The cells were then treated with anti-iNOS and anti-mouse IgG H&L antibodies (Alexa Fluor^®^ 647, Abcam) before mounting using fluorescent mounting medium with DAPI. Confocal images were captured with a fluorescent confocal microscope (Carl Zeiss).

### 2.10. Annexin V-FITC/PI Staining

Apoptotic cell death was assessed by staining the cells using a FITC annexin V apoptosis detection kit with propidium iodide (PI, Biolegend, San Diego, CA, USA) and analyzed with a FACSCalibur flow cytometer (BD Biosciences, San Jose, CA, USA).

### 2.11. Analysis of Cell Cycle Distribution

Cell cycle distribution and DNA contents were determined using PI staining with a PI flow cytometry kit (Abcam) and analyzed with a FACSCalibur flow cytometer (BD Biosciences) and a CytoFLEX flow cytometer (Beckman Coulter, Brea, CA, USA).

### 2.12. Quantitative PCR (qPCR)

Total RNA was extracted using an extraction kit (iNtRON Biotech Inc., Seongnam, Republic of Korea). Subsequently, cDNA synthesis was conducted from the total RNA using a cDNA synthesis kit (Bioneer Corporation, Daejeon, Republic of Korea). The qPCR analysis was performed using Power SYBR^®^ Green Master Mix (Thermo Fisher Scientific, Waltham, MA, USA) in a Real-Time PCR System (Applied Biosystems, Waltham, MA USA), with the following primers: mPD-L1 (NM_021893), For: 5′-TGCTGCATAATCAGCTACGG-3′, Rev: 5′-GCTGGTCACATTGAGAAGCA-3′; mGAPDH (NM_008084), For: 5′-CCAATGTGTCCGTCGTGGATCT-3′, Rev: 5′- GTTGAAGTCGCAGGAGACAACC-3′); hPD-L1 (NM_014143), For: 5′-ATTTGGAGGATGTGCCAGAG-3′, Rev: 5′-CCAGCACACTGAGAATCAACA-3′); and hGAPDH (NM_002046), For: 5′-TCGACAGTCAGCCGCATCTTCTTT-3′, Rev: 5′-ACCAAATCCGTTGACTCCGACCTT-3′. The relative quantity of mRNA was determined through comparative threshold cycle (Ct) analysis.

### 2.13. T Cell-Mediated Cytotoxicity Assay

The T cell-mediated cytotoxicity assay was conducted according to modified methods [30,31,32]. Effector cells, CTLL-2 cells, were co-incubated with target cells, B16F10 cells (20:1 ratio). After incubation with STIG, the release of lactate dehydrogenase (LDH) from supernatants was analyzed using a cell cytotoxicity assay kit (DoGenBio), and the viability of the remaining viable B16F10 cells was assessed using the MTT assay as described earlier. Cytotoxicity was calculated as follows: LDH release (%) = 100 × (A − B − C)/(D − B); where A represents the experimental release value minus the background value (value of assay media), B is the spontaneous release value from B16F10 cells minus the background value (value of assay media), C is the spontaneous release value from CTLL-2 cells (at the basal level) minus the background value (value of assay media), and D is the maximum release value from B16F10 cells minus the value of assay media plus lysis solution. The remaining B16F10 cells were photographed under a microscope.

### 2.14. Statistics

The data are presented as mean ± SEM. All analyses were conducted using IBM SPSS software. Statistical comparisons among multiple groups were performed using one-way analysis of variance (ANOVA) followed by Tukey’s post hoc test. For comparisons between two groups, differences with *p* < 0.05 were considered statistically significant using Student’s unpaired *t*-test.

## 3. Results

### 3.1. STIG Alleviates ROS Levels in B16F10

**Figure 1:**
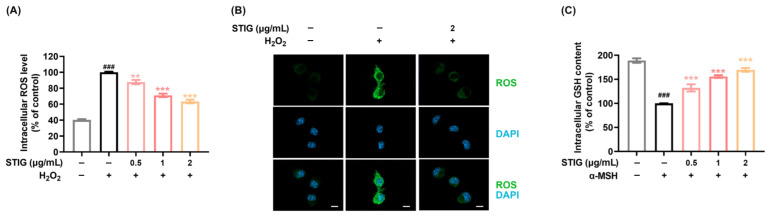
The impact of STIG on ROS production. (**A**) ROS levels were assessed by the addition of H_2_DCFDA. ^###^ *p* < 0.001, compared with the untreated group (blank). ** *p* < 0.01 and *** *p* < 0.001, compared with the H_2_O_2_-treated group (control). (**B**) ROS (green) were observed under a fluorescence microscope (scale bar = 10 µm). (**C**) Analysis of intracellular GSH content was performed as described in Section 2.4. ^###^ *p* < 0.001, compared with the untreated group (blank). *** *p* < 0.001, compared with the α-MSH-treated group (control).

H_2_O_2_ production due to oxidative stress is associated with melanogenesis [33]. Antioxidant capacity is important in down-regulating melanin production [34]. Thus, the antioxidant capacity of STIG was first investigated by measuring intracellular ROS levels induced by H_2_O_2_ in B16F10 melanoma cells. Figure 1A shows that STIG suppressed the ROS levels that were up-regulated by H_2_O_2_ in B16F10 cells. Representative confocal images further illustrate the reduction in intracellular ROS levels following STIG treatment (Figure 1B). In addition, STIG improved the antioxidant protein, GSH, content in α-MSH-treated cells (Figure 1C).

### 3.2. STIG Alleviates Melanogenesis of B16F10

**Figure 2:**
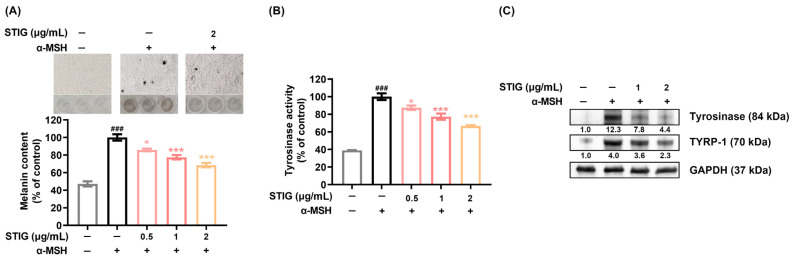
The impact of STIG on melanin content. The cells were pre-treated with STIG and then treated with α-MSH for 48 h. (**A**) Relative melanin levels were determined at 405 nm. Cell images were captured using a bright-field microscope (100× magnification). (**B**) Relative tyrosinase activity was analyzed at 475 nm. ^###^ *p* < 0.001, compared with the untreated group (blank). * *p* < 0.05 and *** *p* < 0.001, compared with the α-MSH-treated group (control). (**C**) Protein levels were assessed using Western blot analysis. Each value below the blot was quantified relative to the untreated group (blank).

We performed a melanin content assay to determine the anti-melanogenesis effects of STIG. The addition of α-MSH to melanocytes induces melanogenesis [9]. As expected, the melanin content increased by α-MSH treatment was markedly suppressed by STIG (Figure 2A). Representative photographs depicting melanin synthesis in each group are provided at the top of Figure 2A. Melanin content is influenced by key melanogenic enzymes in melanocytes, such as tyrosinase and TYRP-1, and is formed through the oxidation of tyrosine by tyrosinase [35]. Thus, we investigated whether STIG modulates tyrosinase activity, and our results clearly demonstrated that STIG decreased α-MSH-induced tyrosinase activity (Figure 2B). STIG also suppressed tyrosinase and TYRP-1 levels (Figure 2C). Furthermore, STIG effectively reduced the melanin content in melan-a cells without causing cellular toxicity (Figure S1).

### 3.3. STIG Alleviates NO Production in B16F10

**Figure 3:**
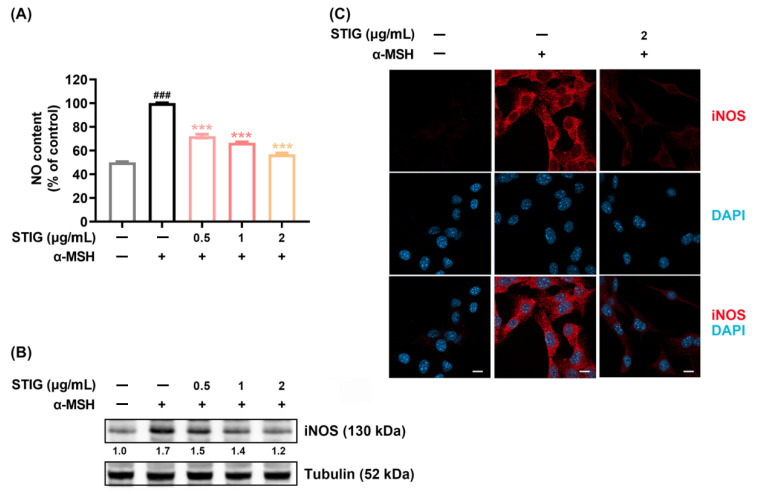
The impact of STIG on NO production. (**A**) Cells were exposed to STIG and then incubated with α-MSH for 48 h. NO levels were determined using Griess reagents. ^###^ *p* < 0.001, compared with the untreated group (blank). *** *p* < 0.001, compared with the α-MSH-treated group (control). (**B**) B16F10 cells treated with STIG were incubated with α-MSH for 24 h. Each expression was analyzed using Western blot analysis. Each value below the blot was quantified relative to the untreated group (blank). (**C**) The iNOS expression was detected by immunofluorescence staining using specific anti-iNOS antibodies (red) under a fluorescence microscope (scale bar = 10 µm).

NO acts as a melanogenesis stimulator by enhancing tyrosinase and TYRP-1 levels and synthesizing melanin pigments in melanocytes [9]. Therefore, we investigated the modulatory effect of STIG on NO production. The NO production was significantly increased by α-MSH. However, this NO production was inhibited when STIG was co-incubated with α-MSH (Figure 3A). We also assessed whether STIG regulates iNOS expression because NO is synthesized by iNOS. As shown in Figure 3B, α-MSH increased the iNOS expression, and this expression was suppressed by STIG. Furthermore, the inhibitory effect of STIG on iNOS levels was confirmed by immunofluorescence staining (Figure 3C).

### 3.4. STIG Induces Apoptotic B16F10 Cell Death

**Figure 4:**
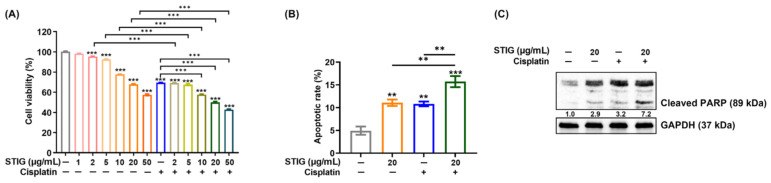
The impact of STIG on apoptosis. (**A**) Cell viability was assessed for B16F10 cells following exposure to various doses of STIG and cisplatin for 48 h. (**B**) Quantification of apoptotic cells was performed through FACS analysis using PI-Annexin V staining. ** *p* < 0.01 and *** *p* < 0.001, compared with the untreated group (blank). (**C**) B16F10 cells were exposed to STIG for 24 h. Protein levels of cleaved PARP were evaluated by Western blot analysis. Each value below the blot was quantified relative to untreated group (blank).

Melanin synthesis and oxidative stress are highly involved in melanoma progression [4]. Thus, we next investigated the effect of STIG on apoptosis in B16F10 cells. A cell viability assay revealed that STIG markedly decreased cell viability at concentrations exceeding 2 μg/mL (Figure 4A). Based on these results, 20 μg/mL of STIG was chosen for subsequent experiments. STIG increased the apoptotic rate, and co-incubation with cisplatin resulted in lower cell viability and higher levels of apoptosis compared with each single group (STIG concentrations above 10 μg/mL, Figure 4A,B). Immunoblots also show higher levels of cleaved PARP expression in the STIG co-culture with cisplatin (Figure 4C). Furthermore, STIG reduced the viability of A375 human melanoma cells, and co-incubation with cisplatin also led to lower cell viability compared with each single group (Figure S2).

### 3.5. STIG Causes Cell Cycle Arrest in B16F10

**Figure 5:**
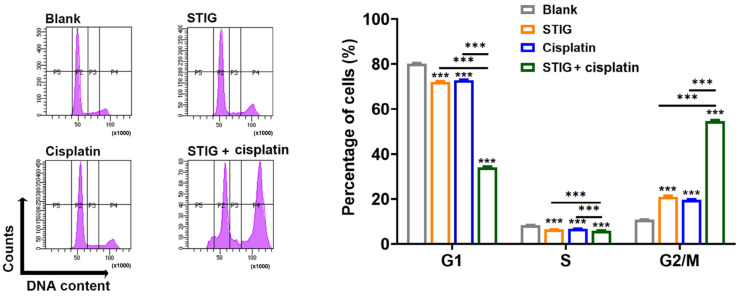
The impact of STIG on cell cycle arrest. The cells were cultured with STIG (20 µg/mL) and cisplatin for 48 h, and cell cycle arrest was measured using flow cytometry. Summary data appear on the right. *** *p* < 0.001, compared with the untreated group (blank).

Inhibition of cell growth is associated with cell cycle progression as well as apoptosis. B16F10 cells treated with STIG exhibited a significant increase in cell cycle arrest at the G2/M phase (Figure 5). Encouragingly, co-incubation with STIG and cisplatin resulted in a dramatic increase in cell cycle arrest at the G2/M phase (Figure 5). When STIG was incubated with anti-cancer agents, nutlin-3a [36] and rapamycin [37], that induce typical G1 arrest, we further investigated whether these combinations induce G1 arrest. Figure S3 demonstrates that co-incubation with STIG and nutlin-3a led to the G1 arrest. However, this combination did not show synergistic effects at the G1 arrest. The co-incubation with STIG and rapamycin did not induce the G1 arrest.

### 3.6. STIG Reduces PD-L1 Expression in B16F10

**Figure 6:**
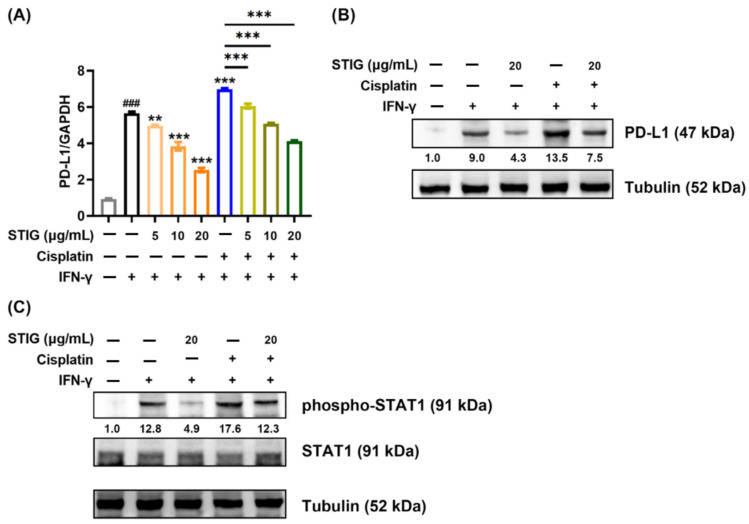
The impact of STIG on PD-L1 levels. Cells treated with STIG or cisplatin were exposed to IFN-γ for 24 h. (**A**) The PD-L1 mRNA and (**B**) protein expression were measured by qPCR and Western blots, respectively. Each value below the blot was quantified relative to the untreated group (blank). ^###^ *p* < 0.001, compared with the untreated group (blank). ** *p* < 0.01 and *** *p* < 0.001, compared with the IFN-γ-treated group. (**C**) Cells treated with STIG or cisplatin were exposed to IFN-γ for 10 min. The protein levels of phospho-STAT1 were measured by Western blots.

PD-L1 expression up-regulates tumor development including melanoma [38,39]. IFN-γ strongly enhances PD-L1 levels in melanoma cells [38,39]. Therefore, we investigated whether STIG regulates IFN-γ-induced PD-L1 levels in B16F10 cells. STIG effectively reduced the PD-L1 mRNA levels increased by IFN-γ (Figure 6A) and suppressed the IFN-γ-induced PD-L1 protein levels (Figure 6B). Interestingly, cisplatin contributes to PD-L1 expression, which provides an escape route for tumor cells to evade immune detection [40]. In accordance with previous reports that cisplatin increases PD-L1 levels in cancer cells, Figure 6A–C shows that cisplatin significantly increased IFN-γ-induced PD-L1 levels, and STIG effectively decreased these levels. IFN-γ-induced PD-L1 expression is dependent on the transcription factor STAT1 [39,41]. IFN-γ induces PD-L1 transcription in tumor cells by increasing the acetylation of the PD-L1 promoter and the phosphorylation of STAT1 at Tyr701 [41]. Thus, we investigated whether STIG regulates STAT1 phosphorylation augmented by IFN-γ or both IFN-γ and cisplatin. As expected, IFN-γ or both IFN-γ and cisplatin augmented phospho-STAT1 levels, and STIG significantly reduced the phospho-STAT1 levels augmented by IFN-γ or both IFN-γ and cisplatin (Figure 6C). These results indicate that STIG reduces PD-L1 levels induced by IFN-γ by down-regulating STAT1 signaling. Furthermore, we confirmed that STIG reduced the PD-L1 mRNA levels increased by IFN-γ or both IFN-γ and cisplatin in A375 human melanoma cells (Figure S4).

### 3.7. STIG Regulates T Cell-Mediated Cytotoxicity against B16F10

**Figure 7:**
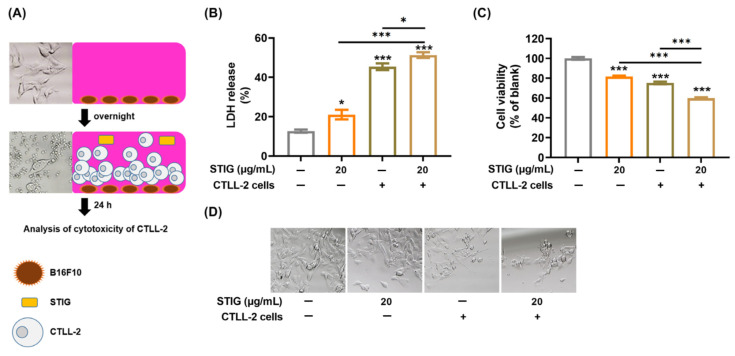
The impact of STIG on the cytotoxicity of CTLL-2 cells. (**A**) The image is a schematic diagram used to assess the cytotoxicity of CTLL-2 cells. (**B**) Cytotoxicity was assessed via an LDH release assay. (**C**) Cell viability was measured via an MTT assay. * *p* < 0.05 and *** *p* < 0.001, compared with the untreated group (blank). (**D**) Images of the remaining viable B16F10 cells were captured under a bright-field microscope (100× magnification).

PD-L1 expression on cancer cells inhibits CD8(+) T cell function [13]. Thus, we finally investigated whether STIG augments the CD8(+) T cell function using CTLL-2 cells. We utilized a co-culture system by exposing B16F10 cells to CTLL-2 cells, followed by treatment with STIG (Figure 7A). Figure 7B shows that STIG significantly promoted the cytotoxicity of CTLL-2 cells against B16F10 cells by increasing LDH release. Subsequently, STIG further suppressed the cell viability of B16F10 cells reduced by CTLL-2 cells (Figure 7C,D).

## 4. Discussion

**Figure 8:**
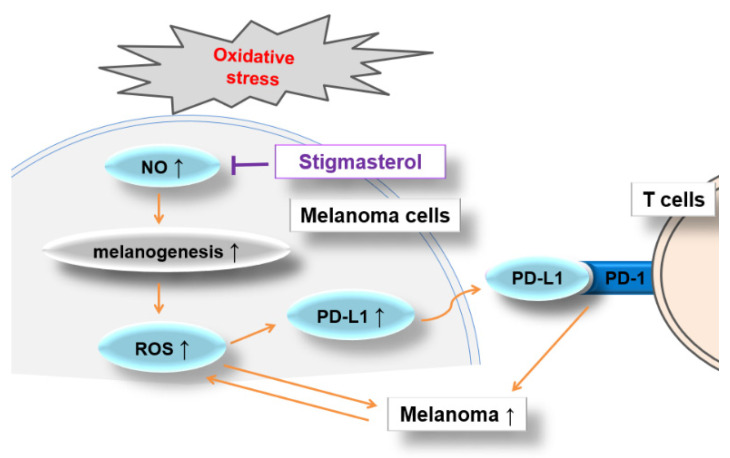
Schematic depicting the anti-melanoma effect of STIG through the down-regulation of ROS and PD-L1 levels in melanoma cells.

The incidence of the most lethal cutaneous melanoma is increasing worldwide, and this trend is expected to continue according to projections from the WHO [42,43]. While the development of targeted therapies and chemotherapy has greatly increased patient survival rates, long-term treatments are limited because of side effects and rapid resistance [44,45,46]. The clinical success of immune checkpoint blockade for melanoma has validated the effectiveness of treatment in reactivating the immune system for effectively targeting melanoma [47]. Pembrolizumab, an anti-PD-1 antibody, has extended the survival of patients with advanced melanoma [48]. Hence, Iwai et al. suggested that blocking the interaction between PD-1 and PD-L1 offers a promising strategy for specific tumor immunotherapy [49]. However, Huang and Zappasodi noted that despite the success of immune checkpoint blockade, efficacy has plateaued, necessitating drug discovery efforts [47]. Therefore, there is a need to explore new agents to overcome the limitations of current treatment strategies. We demonstrated that STIG exhibits potent activity against melanoma cells by strongly inducing apoptosis in B16F10 cells, down-regulating melanogenesis, and reducing ROS production. Furthermore, STIG treatment significantly attenuated PD-L1 levels via STAT1 signaling and PD-L1-mediated immunotherapy resistance of cisplatin. This implies that STIG may be a promising agent to treat melanoma (Figure 8).

Previous studies have suggested the use of antioxidants for melanoma prevention or treatment [43,50]. The skin is continuously exposed to exogenous oxidative stress, with melanocytes particularly vulnerable due to the melanogenesis process, which generates ROS [51]. Oxidative stress is related to all stages of melanoma development [52]. ROS are closely associated with the immunosuppressive tumor microenvironment by driving cancer progression, regulating PD-1 expression, and inhibiting T cell function [53]. Melanogenesis in melanocytes results in higher levels of intracellular ROS, which can lead to an increase in melanoma susceptibility [54]. Cutaneous cancer cells are readily exposed to oxidative environments, with ROS having a dual role in cancer cells [55]. At toxic doses, ROS induce apoptosis and kill cancer cells [55,56]. However, H_2_O_2_ as a typical ROS molecule can promote abnormal proliferation and metastasis of tumor cells by up-regulating oxidative stress-related signaling [55]. Lowering local ROS levels enhances the immune response to melanoma by inhibiting PD-L1 signaling in vivo [53,57]. Quercetin is excellent for strengthening antioxidant defenses through H_2_O_2_ removal and has a strong anti-cancer effect against skin cancer [58]. Yu and Wang emphasized the importance of alleviating the immunosuppressive tumor microenvironment by removing ROS from the tumor site [53]. In addition, as a major antioxidant, GSH prevents the development of various diseases through the removal of ROS [59]. As with previous evidence, we found that STIG down-regulates H_2_O_2_-induced ROS and IFN-γ-induced PD-L1 levels in melanoma cells while increasing GSH levels. Therefore, we propose that STIG may exert anti-tumor effects on melanoma by targeting ROS as a critical factor.

Both α-MSH and NO in melanocytes promote melanogenesis by increasing tyrosinase activity [60]. In addition, Yang et al. suggested that targeting NO signaling may be therapeutic and preventive because melanoma cells are characterized by high expression of NO synthase, and NO is deeply involved in increasing invasion and proliferation of melanoma cells [61]. Inhibiting iNOS and iNOS-derived NO levels has been shown to reduce melanoma growth [62,63,64,65]. Moreover, NO induces PD-L1 expression in cancer, and reducing NO signaling suppresses the PD-1/PD-L1 pathway by lowering PD-L1 levels [66]. Consistent with previous findings, we have demonstrated that STIG inhibits α-MSH-induced melanin, tyrosinase activity, and iNOS, and NO levels in melanoma cells. Thus, we propose that STIG might suppress melanoma development by down-regulating melanogenesis and NO signaling. Furthermore, we observed a decrease in melanin content by STIG in melan-a cells, indicating that STIG has a regulatory effect on melanogenesis in both normal melanocytes and melanoma.

Cisplatin and STIG alone induced cell death, and flow cytometry results supported this by showing that they induced cell cycle arrest at the G2/M phase. Although the sub-G1 population is an indicator of apoptosis [67], no sub-G1 peak was observed by STIG in the present study. However, the absence of the sub-G1 population does not necessarily mean there is no apoptosis in the cell population and sub-G1 population cannot be used as a definitive marker of apoptosis without additional specific tests [39,67]; DNA fragmentation, which is the hallmark of apoptosis, provides a basis for flow cytometric assays including cell cycle analysis to identify apoptotic cells [67]. The sub-G1 population based on the analysis of DNA fragmentation may contain physically disintegrated cells or chromatin aggregates, masquerading the presence of apoptotic cells [67]. Cell cycle analysis of apoptosis may not clearly distinguish between sub-G1 populations due to other forms of cell death, debris, or intact single cells [68]. In addition, the sub-G1 population can encompass both apoptotic and necrotic cells [69,70,71,72]. The previous reports have identified the anti-cancer potential of anti-cancer agents by revealing that they induce cell death only through G2/M arrest [39,73]. Thus, we suggest that STIG, which induces cell cycle arrest at the G2/M phase, exerts pro-apoptotic activity against melanoma cells, providing new insights into the anti-melanoma effects of STIG. Additionally, given that cisplatin does not induce G1 arrest [74], we further determined that the combination of STIG with nutlin-3a, which induces G1 arrest, led to G1 arrest, although this combination did not show a synergic effect on the G1 arrest. STIG displayed a synergistic effect on cell cycle arrest when combined with cisplatin, which induces G2/M arrest. Therefore, these results suggest that STIG is more effective when administered as a combination therapy with anti-cancer drugs that induce G2/M arrest. However, in order to administer STIG as a combination therapy, further investigation is needed with various cell-cycle-specific anti-cancer drugs.

Cisplatin promotes PD-L1 expression in tumors, leading to resistance to immune therapy [40,75]. Thus, previous studies have explored combination therapy involving cisplatin and immune checkpoint inhibitors [75,76]. Selective antagonists of iNOS have also been shown to inhibit chemoresistance in human melanoma to cisplatin [65]. This study found that STIG not only inhibited the increase in PD-L1 levels induced by cisplatin but also reduced iNOS and NO levels. Moreover, the combination of STIG with cisplatin resulted in more effective apoptosis of melanoma cells. It is possible that STIG might exert anti-melanoma effects by reducing the PD-L1 levels increased by cisplatin through the down-regulation of NO signaling. STIG could be applied in combination with cisplatin to address chemotherapy and immunotherapy resistance. Therefore, we propose that combination therapy with STIG and cisplatin may enhance treatment efficacy by creating conditions in which cisplatin induces melanoma cell death. However, other mechanisms may also contribute to the anti-tumor effects of this combination involving PD-1/PD-L1 blockade. More research is required to determine the mechanisms by which STIG down-regulates PD-1/PD-L1 pathways following cisplatin treatment.

Immunogenic cell death induced by anti-cancer drugs is becoming increasingly recognized as an important cancer treatment strategy, particularly when combined with anti-cancer immunotherapy [77]. This form of cell death activates tumor-specific CD8(+) T cell-mediated immune reactions, leading to systemic effects, as observed when radiotherapy is combined with checkpoint blockers [77]. The combination of chemotherapy and immunotherapy targeting PD-L1/PD-1 has shown promise in improving anti-tumor responses by triggering immune-reactivation via CD8(+) T cells [76]. The co-incubation of PD-1-expressing CTLL-2 cells, which are CD8(+) T cells and melanoma cells, has been used in cancer research [78,79]. Our findings indicate that STIG enhances the killing effect of CTLL-2 cells on melanoma cells, suggesting its potential application in inducing immunogenic cell death in melanoma. However, the precise mechanisms underlying the effector function of cytotoxic T cells need to be elucidated in the anti-tumor activity of STIG.

## 5. Conclusions

We found that STIG possesses anti-melanoma properties by inducing antioxidant effects and down-regulating PD-L1 expression. Moreover, STIG reversed the immune-suppressive effects of cisplatin, indicating that combination therapy with STIG and cisplatin more effectively induces melanoma cell death. This study highlights STIG as an effective approach for melanoma treatment strategies.

## Data Availability

The data sets used and/or analyzed during the current study are available from the corresponding author.

## Supplementary Materials

The following supporting information can be downloaded at: https://www.mdpi.com/article/10.3390/antiox13030380/s1, Figure S1: The impact of STIG on melanin content in melan-a cells; Figure S2: The impact of STIG on the cell viability of A375 cells; Figure S3: The impact of the co-incubation with STIG, nutlin-3a, or rapamycin on cell cycle arrest; and Figure S4: The impact of STIG on PD-L1 mRNA levels in A375 cells.
