# Supplementary material for: Stigmasterol Exerts an Anti-Melanoma Property through Down-Regulation of Reactive Oxygen Species and Programmed Cell Death Ligand 1 in Melanoma Cells
Source: Antioxidants (Basel). 2024 Mar 21;13(3):380. doi: 10.3390/antiox13030380 (PMC10967816; doi:10.3390/antiox13030380)
Supplement: Supplementary file 1 [file antioxidants-13-00380-s001.zip › Supplementary File 1.pdf]

Supplementary data

Stigmasterol Exerts an Anti-Melanoma Property through Down-Regulation of ROS and PD-L1 in Melanoma Cells

Na-Ra Han [1,2], Tae-Hoo Yi [3], Hi-Joon Park [4], Seong-Gyu Ko [2,5] and Phil-Dong Moon [6,*]

Supplementary Figures

Figure S1. The impact of STIG on melanin content in melan-a cells

Figure S2. The impact of STIG on the cell viability of A375 cells

Figure S3. The impact of the co-incubation with STIG, nutlin-3a, or rapamycin on cell cycle arrest

Figure S4. The impact of STIG on PD-L1 mRNA levels in A375 cells

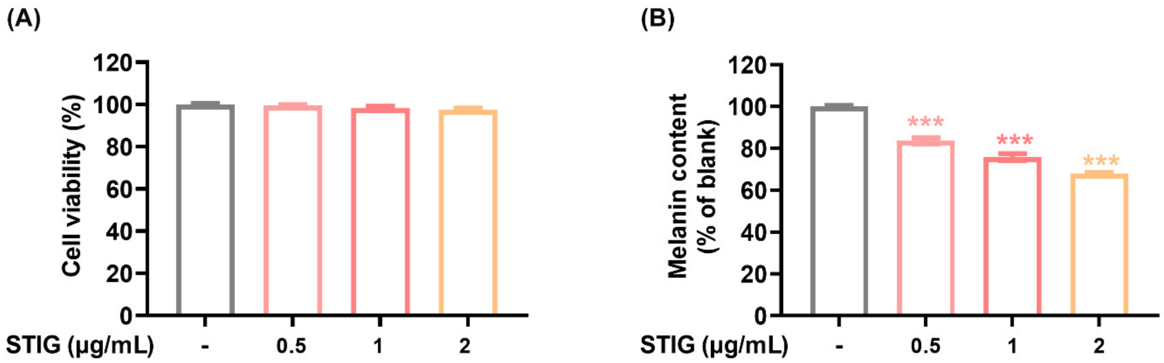

Figure S1. The impact of STIG on melanin content in melan-a cells. **(A)** Cell viability was assessed for melan-a cells following exposure to various doses of STIG for 48 h. (B) The relative melanin levels were determined at 405 nm. $^{***}p < 0.001$, compared with the untreated group (blank).

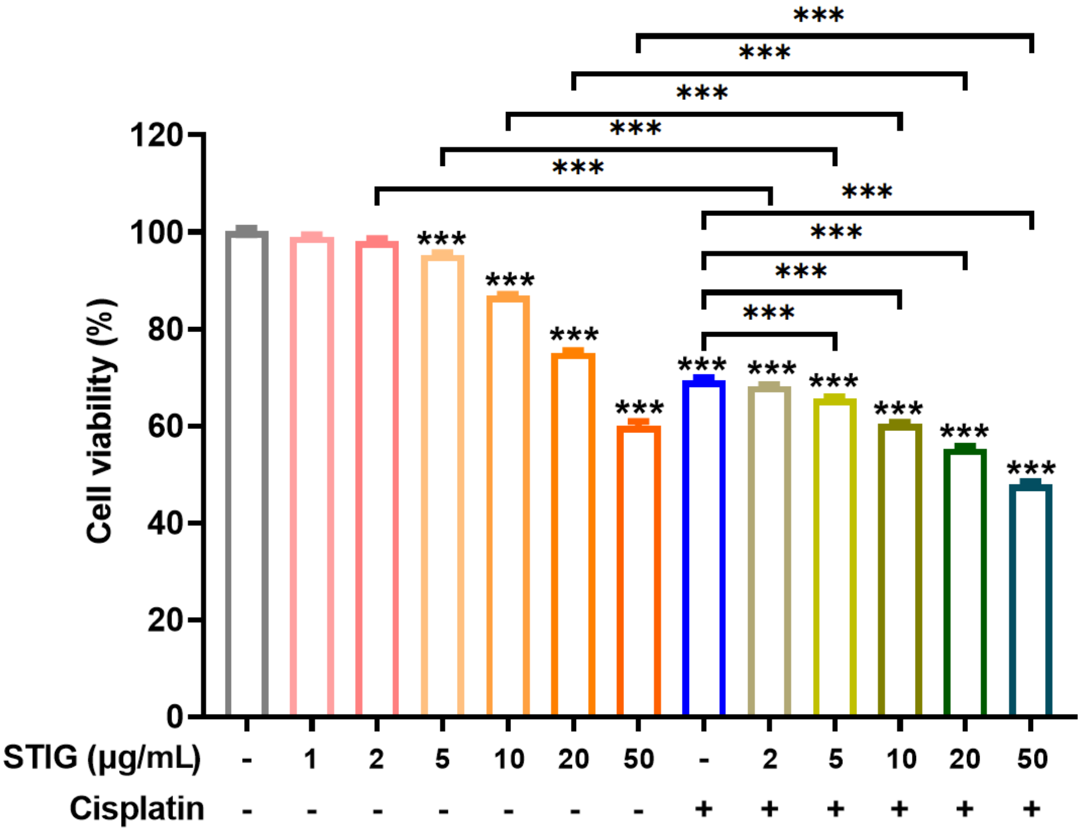

Figure S2. The impact of STIG on the cell viability of A375 cells. Cell viability was assessed for A375 cells following exposure to various dose of STIG and cisplatin for 48 h. $^{***}p < 0.001$, compared with the untreated group (blank).

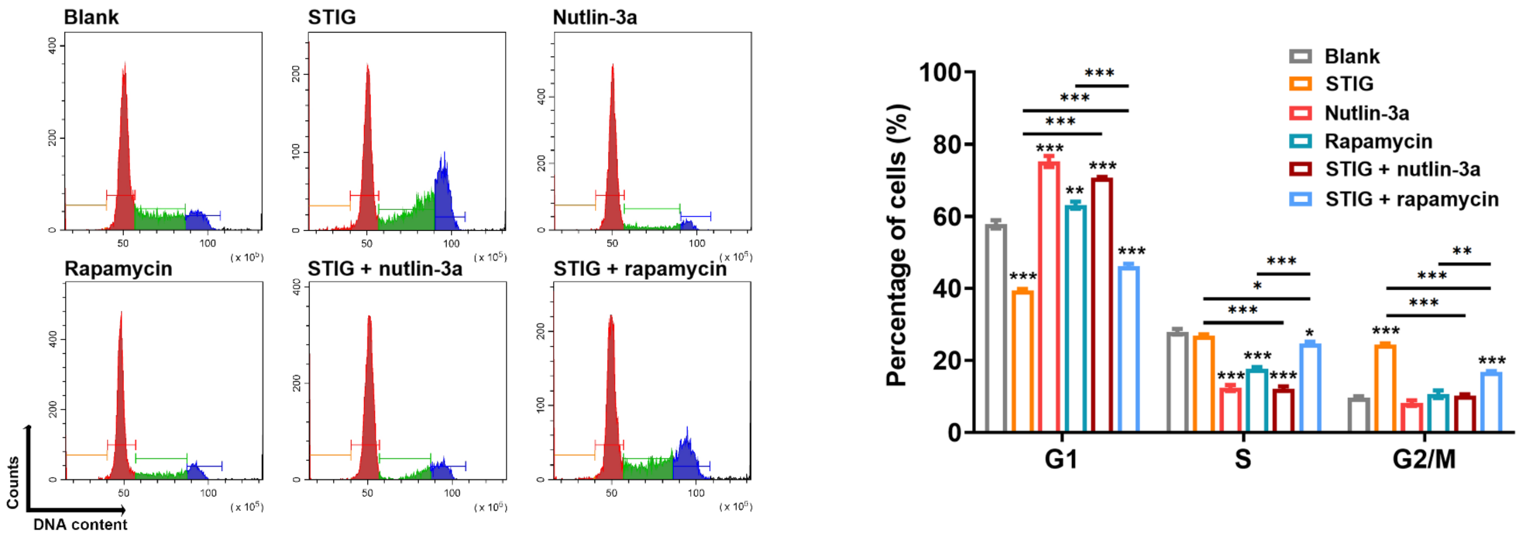

Figure S3. The impact of the co-incubation with STIG, nutlin-3a, or rapamycin on cell cycle arrest. Cell cycle arrest was measured using flow cytometry. Summary data appears on the right. $^{*}p < 0.05$, $^{**}p < 0.01$, and $^{***}p < 0.001$, compared with the untreated group (blank).

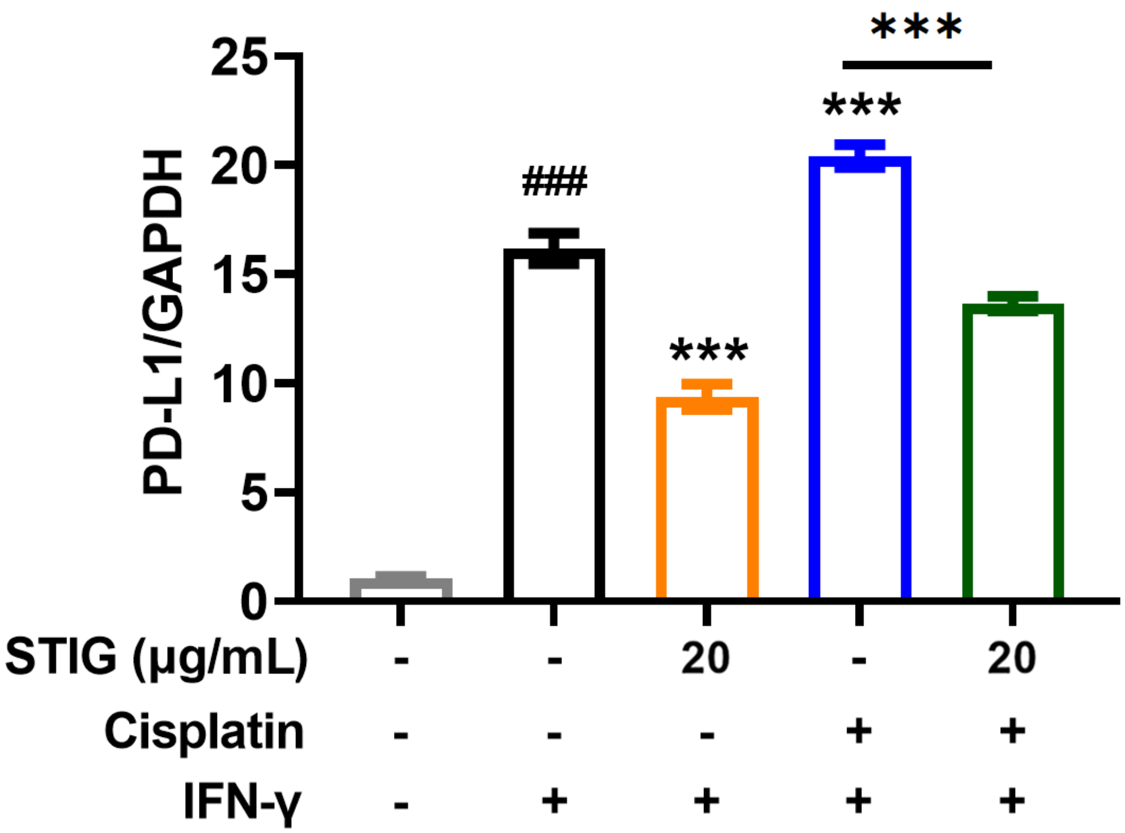

Figure S4. The impact of STIG on PD-L1 mRNA levels in A375 cells. The cells treated with STIG or cisplatin were exposed to IFN-γ for 24 h. The PD-L1 mRNA level was measured by qPCR. $^{###}p < 0.001$, compared with the untreated group (blank). $^{***}p < 0.001$, compared with the IFN-γ-treated group (control).
